# Supplementary material for: The genome sequence of the Southern White Admiral, Limenitis reducta (Staudinger, 1901) (Lepidoptera: Nymphalidae)
Source: Wellcome Open Res. 2026 Feb 9;11:97. [Version 1] doi: 10.12688/wellcomeopenres.25845.1 (PMC13129534; doi:10.12688/wellcomeopenres.25845.1)
Supplement: Peer review history

# Reviewer report: Reviewer response for version 1

**DOI:** 10.21956/wellcomeopenres.28446.r154174

**Published:** 2026-05-06

**Authors:** Yukun Sun

**Affiliations:** 1 The Field Museum, Chicago, Illinois, USA

This Data Note presents a chromosome-level, haplotype-resolved genome assembly for a female *Limenitis reducta*specimen. The work was produced through Project Psyche and the Wellcome Sanger Institute Tree of Life Programme. The authors used PacBio HiFi reads, Arima-HiC data, Hifiasm, YaHS, manual curation, MitoHiFi, BlobToolKit, Merqury.FK, BUSCO, and Ensembl annotation. The data are deposited under PRJEB79421. The article fits the Wellcome Open Research model, where review should assess academic merit and validity rather than novelty. Overall, the assembly is strong and well documented. Haplotype 1 spans 397.72 Mb across 164 scaffolds. Most sequence, 98.92%, is assigned to 31 chromosomal pseudomolecules. The reported QV values are high. BUSCO completeness is also high. The Hi-C map, Merian-element painting, MerquryFK plot, and BlobToolKit plots support the assembly quality. The software version table also improves reproducibility. In my view, the article is scientifically sound. I do not see issues that require new analysis before the article can be considered valid. However, several clarifications would make the paper more useful for readers and downstream users.

 Major points

 1. Explain the unresolved region on chromosome 22. The text says contigs from 3.5 Mb to 9.2 Mb have an unclear order. That is about 5.7 Mb on an 11.17 Mb chromosome. So roughly half of the chromosome is uncertain. Users doing synteny, recombination, or structural-variant work need to know why. Likely causes are weak Hi-C signal, repeats, heterochromatin near the centromere, or haplotype switching. The authors should state the most likely cause. If the cause is unclear, they should say so. One or two sentences in the Assembly curation section would be enough. A short note on whether ONT reads or optical mapping could fix the region would also help.

 2. Make the reason for sequencing this species clearer **. **The Background has good ecological detail. But the reason for choosing this species stays implicit. One short sentence in the Abstract or Background would help. For example, *L. reducta* is Least Concern globally but critically endangered in Germany. That contrast makes it useful for regional conservation genomics. The genome also fits comparative chromosome work in Nymphalidae.

 3. Clarify the k-mer completeness benchmark. Table 4 lists ≥95% as the target. The reported values are 88.26% for haplotype 1 and 82.98% for haplotype 2. Only the combined value (99.33%) meets the target. This split is normal for phased diploid assemblies. But the table can read as if the benchmark is missed. A short footnote, or one extra clause in the text, would fix this.

 Minor points

 4. Note the limits of automated annotation. Ensembl reports 12,058 protein-coding genes and 2,402 non-coding genes. There is no species-specific RNA-seq input. So UTRs, alternative isoforms, and lineage-specific genes may be incomplete. One short sentence pointing readers to this would help downstream users.

 5. Consider releasing curation artifacts. The raw Hi-C reads are public, which is good. But Pretext maps or curation BAMs would help others re-curate chromosome 22. A version tag on the rapid-curation repo would also help reproducibility.

 6. Style and formatting. Use "haplotype 1" and "haplotype 2" everywhere. Avoid switching to "primary" or "secondary" outside the accession names. Numbers such as "12 058", "22 915", and "1 400 m" use thin spaces and render unevenly across viewers. Pick one format and use it throughout.

 7. Small wording. "The larvae hibernates" should read "The larva hibernates" or "Larvae hibernate".

 I did not re-run the assembly, scaffolding, contamination screen, BUSCO, Merqury.FK, BlobToolKit, MitoHiFi, or Ensembl pipelines. I did not inspect the raw reads. I did not test single gene models. My review is based on the text, figures, tables, methods, software list, and public accessions.

Are sufficient details of methods and materials provided to allow replication by others?

Yes

Is the rationale for creating the dataset(s) clearly described?

Yes

Are the datasets clearly presented in a useable and accessible format?

Yes

Are the protocols appropriate and is the work technically sound?

Yes

Reviewer Expertise:

Genome assembly, comparative genomics, phylogenomics, bioinformatics, long-read sequencing, chromosome-level assembly assessment, genome annotation, and evolutionary genomics.

I confirm that I have read this submission and believe that I have an appropriate level of expertise to confirm that it is of an acceptable scientific standard, however I have significant reservations, as outlined above.

# Reviewer report: Reviewer response for version 1

**DOI:** 10.21956/wellcomeopenres.28446.r154169

**Published:** 2026-04-30

**Authors:** Anwesha Das

**Affiliations:** 1 Sanaka Educational Trust’s Group of Institutions (SETGOI), West Bengal, India

The manuscript entitled as "The genome sequence of the Southern White Admiral, *Limenitis reducta* (Staudinger, 1901) (Lepidoptera: Nymphalidae)" presents a chromosome‑level, haplotype‑resolved genome assembly for the Southern White Admiral butterfly ( *Limenitis reducta*), generated as part of Project Psyche and the Wellcome Sanger Institute Tree of Life programme. Using PacBio HiFi long‑read sequencing and Hi‑C scaffolding, the authors assembled two haplotypes, with haplotype 1 curated to 31 chromosomal pseudomolecules (29 autosomes plus W and Z sex chromosomes). The final assembly totals 397.72 Mb for haplotype 1, with very high consensus quality (QV ≈ 63), strong BUSCO completeness (98.6%), and extensive chromosomal assignment (98.92%). The authors additionally report assembly metrics in line with Earth BioGenome Project reference standards, include a fully assembled mitochondrial genome (15.18 kb), and provide Ensembl-based genome annotation identifying 12,058 protein‑coding genes. The manuscript largely follows established Data Note conventions, providing extensive methodological detail, clear quality assessment, and open access to all underlying sequencing data and assemblies. Overall, the work constitutes a high‑quality genomic resource for Lepidoptera research, conservation genomics, and comparative chromosome evolution studies. This is a technically strong and clearly presented genome resource that meets or exceeds current standards for reference-quality assemblies in insects. The assembly quality metrics, methodological transparency, and data availability are excellent. The manuscript aligns well with expectations for a Data Note and will be valuable to a wide range of users. The study is scientifically sound; however, a small number of clarifications and contextual improvements would significantly strengthen the manuscript’s usefulness and transparency, particularly for non-specialist readers or downstream users.

**Major Points**

 1. Explicit explanation of the unresolved region on chromosome 22

 The manuscript notes that: “Contigs along chromosome 22 between 3.5 Mb and 9.2 Mb have uncertain order and orientation.” This is a notable exception in an otherwise chromosome‑complete assembly, yet no explanation is provided.

 Unresolved regions may affect downstream analyses (e.g., recombination, structural variation, gene order, or comparative synteny studies). Users need to understand whether the uncertainty is due to biological features (e.g., repeats, heterochromatin), technical limitations, or insufficient Hi‑C signal.

 Briefly explain why this region remains unresolved. For example: Low Hi‑C contact density, Conflicting Hi‑C signals, High repeat content or structural complexity, Potential misassembly between haplotypes. If the reason is unclear, state this explicitly. Indicate whether future resequencing or alternative scaffolding approaches could resolve the region. This can be addressed in one or two sentences in the Genome sequence report or Assembly curation sections.

 2. Clearer rationale for generating this genome in the abstract or background

 The rationale for sequencing *L. reducta* is implicit (Project Psyche, biodiversity genomics) but only briefly stated. Data Notes serve a broad readership. Explicit rationale helps users understand the biological, ecological, or evolutionary relevance of the species and dataset. Add one concise sentence to the abstract or background clarifying the motivation, e.g.: Its biogeographic distribution, Conservation relevance (e.g. regional endangerment), Importance for Lepidoptera chromosome evolution. This does not require new analyses, only contextual framing.

 3. Brief contextualisation of chromosome number and sex chromosomes

 The assembly reports 29 autosomes plus W and Z sex chromosomes, but this is not contextualised relative to other *Limenitis* or Nymphalidae species. Given the inclusion of Merian element painting and the relevance of chromosome evolution in Lepidoptera, readers would benefit from minimal comparative context. Add one short sentence noting whether this karyotype is typical or notable within: *Limenitis*, Nymphalidae, Lepidoptera more broadly. No comparative analysis is required-just a statement of expectation or consistency.

**Minor Points and Suggestions**

 1. Terminology consistency

 Ensure consistent use of: “haplotype 1 / haplotype 2” vs. “primary / secondary assembly”. This avoids confusion for non-specialist readers.

 2. Gene annotation interpretation

 The Ensembl annotation statistics are clearly reported, but a short sentence stating that annotation follows standard automated Ensembl pipelines (with their known strengths/limitations) would be helpful for users interpreting gene models.

 3. Minor stylistic edits

 A careful proofread could eliminate a small number of typographical issues (e.g. spacing around units such as “1 400 m” or “22 915”).

 I did not independently re‑run assembly or annotation pipelines. I did not directly inspect raw sequencing reads or validate contamination filtering beyond the reported BlobToolKit outputs. Functional accuracy of individual gene models was not assessed (appropriate for a Data Note).

 These limitations are inherent to manuscript‑based peer review and do not detract from the technical quality presented.

Are sufficient details of methods and materials provided to allow replication by others?

Yes

Is the rationale for creating the dataset(s) clearly described?

Yes

Are the datasets clearly presented in a useable and accessible format?

Yes

Are the protocols appropriate and is the work technically sound?

Yes

Reviewer Expertise:

Computational chemistry, medicinal chemistry, pharmacology, assays.

I confirm that I have read this submission and believe that I have an appropriate level of expertise to confirm that it is of an acceptable scientific standard, however I have significant reservations, as outlined above.

# Reviewer report: Reviewer response for version 1

**DOI:** 10.21956/wellcomeopenres.28446.r154173

**Published:** 2026-04-29

**Authors:** Ariel Chipman

**Affiliations:** 1 Hebrew University of Jerusalem Department of Ecology Evolution and Behavior (Ringgold ID: 549289), Jerusalem, Jerusalem District, Israel

The authors report a chromosome-level genome sequence of the nymphal butterfly *Limenitis reducta* (White Admiral), from a female specimen collected in the mountains of Georgia. The sequencing and assembly follow the standard Tree of Life protocols and guidelines. The analysis of the sequenced genome shows a high-quality assembly, with very high completeness scores and BUSCO scores. 

 The written report is clear and concise as expected from this type of article. I have no comments or suggestions for change.

Are sufficient details of methods and materials provided to allow replication by others?

Yes

Is the rationale for creating the dataset(s) clearly described?

Yes

Are the datasets clearly presented in a useable and accessible format?

Yes

Are the protocols appropriate and is the work technically sound?

Yes

Reviewer Expertise:

Arthropod evolution

I confirm that I have read this submission and believe that I have an appropriate level of expertise to confirm that it is of an acceptable scientific standard.

# Reviewer report: Reviewer response for version 1

**DOI:** 10.21956/wellcomeopenres.28446.r147845

**Published:** 2026-03-25

**Authors:** Stuart J.E. Baird

**Affiliations:** 1 Academy of Sciences of the Czech Republic, Národní, Staré Město, Czech Republic

The report largely conforms to the excellent DToL template. The rationale for creating the dataset is only fleetingly mentioned in the abstract. Unusually for DToL, there is a clearly un-assembled region: "Contigs along chromosome 22 between 3.5 Mb and 9.2 Mb have uncertain order and orientation." The reader should be told why, or if the reason is unclear, the reader should be told why it is unclear.

Are sufficient details of methods and materials provided to allow replication by others?

Yes

Is the rationale for creating the dataset(s) clearly described?

Yes

Are the datasets clearly presented in a useable and accessible format?

Yes

Are the protocols appropriate and is the work technically sound?

Yes

Reviewer Expertise:

Admixture genomics

I confirm that I have read this submission and believe that I have an appropriate level of expertise to confirm that it is of an acceptable scientific standard.
